# Age-related differences in activities of daily living among older Chinese adults

**DOI:** 10.1038/s41598-025-18163-y

**Published:** 2025-10-03

**Authors:** Cheng-Hao Chang, Fang Wang, Kai-Lun Yang, Li-Qin Wang, Lan-Xiang Wang, Nan Peng

**Affiliations:** 1 The Eighth Medical Center of Chinese PLA General Hospital, Beijing, China; 2 Department of Rehabilitation Medicine, The Second Medical Center, National Clinical Research Center for Geriatric Diseases, Chinese PLA General Hospital, Beijing, China; 3 China National Center for Biotechnology Development, Beijing, 100039 China; 4 Graduate School, Chinese PLA General Hospital, Beijing, China

**Keywords:** Activities of daily living, Functional decline, Frailty, Aging, China health and retirement longitudinal study, Cross-Sectional study, Risk factors, Epidemiology, Disability, Health occupations

## Abstract

The decline in activities of daily living (ADL) significantly impacts independence and quality of life among older adults. This study used data from the China Health and Retirement Longitudinal Study (CHARLS) to conduct a cross-sectional analysis of 4,751 functionally impaired older Chinese adults aged ≥ 60 years with Modified Barthel Index (MBI) scores < 100. Participants were stratified into seven age groups (60–64, 65–69, 70–74, 75–79, 80–84, 85–89, and ≥ 90 years), and simple linear models were used to analyze age-related differences across 12 ADL domains. Results revealed three patterns of functional decline: (1) basic self-care activities (eating, getting up) remained relatively stable across age groups; (2) mobility functions (walking, stair climbing) showed progressive decline, with walking demonstrating continuous deterioration across multiple age transitions; and (3) personal care activities (bathing, grooming, toileting, dressing) showed significant decline primarily between ages 75–84. Gender-stratified analysis identified female advantages in self-care abilities and age-related changes in walking performance. The 75–84 years period emerged as a critical transition, with six functional domains showing significant deterioration. These findings suggest that interventions targeting mobility preservation and targeted interventions during the late 70s to early 80s may help delay functional decline and maintain independence in older adults.

**Supplementary Information:**

The online version contains supplementary material available at 10.1038/s41598-025-18163-y.

## Introduction

Population aging has emerged as a pervasive global trend, presenting substantial challenges for individuals, families, and societies^1^. A central feature of the aging process is the progressive decline in activities of daily living (ADL), a deterioration that profoundly influences older adults’ quality of life, independence, and overall functional health^2^. The The Modified Barthel Index (MBI) is a robust and widely adopted indicator for assessing ADL functional capacity^3^. Notably, declines in ADLs are closely intertwined with a range of aging-related health conditions, including frailty—a geriatric syndrome characterized by diminished physiological reserves and heightened vulnerability to adverse outcomes^4^. Frailty is a major risk factor for disability and mortality in older adults^5^, as it often manifests as reduced physical function, compromised ADLs, and an increased risk of institutionalization. Understanding ADL difference patterns across age groups is essential for preserving functional health and identifying critical intervention periods.

A functional decline in older adults often manifests as reduced mobility, compromised self-care abilities, and gait impairments^6^. Such decrements are underpinned by multifactorial changes—both physiological and psychological—including musculoskeletal weakness, impaired balance, and cognitive deterioration. These factors collectively contribute to frailty increase and elevate the risk of disability^7^. Existing evidence suggests that the onset and rate of ADL decline differ by functional domain, with some activities (e.g., walking, stair climbing) showing earlier or more pronounced deterioration than others do^8^. Investigating these domain-specific patterns can shed light on the underlying determinants of frailty and functional decline, offering valuable insights into the design of timely and targeted interventions. Moreover, understanding the age-specific patterns of ADL differences decline may help identify optimal time windows for frailty prevention strategies, thereby mitigating disability and enhancing quality of life for older adults.

Despite increasing recognition of these issues, most current evidence derives from institutionalized or clinical populations rather than community-dwelling older adults. Moreover, few longitudinal or cross-sectional studies have examined these patterns in functionally diverse, community-based cohorts. Many investigations emphasize aggregate ADL scores, potentially overlooking nuanced age-related changes in specific functional subdomains. To address these gaps, this study leverages the China Health and Retirement Longitudinal Study (CHARLS)—a nationally representative dataset—to investigate age-related ADL differences in community-dwelling older Chinese adults using a cross-sectional design^9^. While a cross-sectional analysis cannot directly reveal individual patterns of differences over time, the systematic examination of differences across age groups provides important insights into patterns of functional decline.

By identifying the functional domains most susceptible to decline and characterizing their age-related patterns of difference, this study aims to provide a foundation for age-specific, evidence-based strategies for preventing frailty and functional decline while informing the timing of interventions to maximize their efficacy.

## Methods

### Research design and data source

This cross-sectional study utilized data from the China Health and Retirement Longitudinal Study (CHARLS), which provided publicly available information from four survey waves (2011, 2013, 2015, and 2018)^9^. The CHARLS is a nationally representative survey of Chinese adults aged ≥ 45 years. The survey captures comprehensive information on demographics, health status, lifestyle factors, physical functioning, and socioeconomic conditions. The sampling strategy employed a multistage, stratified, probability‒proportional‒to‒size approach to ensure broad geographic and demographic representation of the older Chinese population.

### Handling of repeated measurements

Given that 42.8% of participants (*n* = 2034) appeared in multiple survey waves, creating 7,676 total observations from 4,751 unique individuals, we implemented a random sampling strategy. To satisfy the fundamental assumption of independence required for conventional statistical analyses, we randomly selected one observation per participant for those with repeated measurements. Specifically, we set a random seed (seed = 123) to ensure reproducibility and randomly selected one measurement from each participant’s multiple survey wave data. This deduplication process reduced the dataset from 7,676 observations to 4,751 independent participants, removing 2,925 repeated observations while maintaining sample representativeness through the randomization approach.

### Inclusion and exclusion criteria

This study specifically targeted older adults with varying degrees of functional impairment, thus defining the inclusion criteria as “adults aged ≥ 60 years with MBI < 100.” This criterion was deliberately chosen based on the research objective, which focused on analyzing age-related patterns of differences in ADL domains among older adults who had already experienced functional impairment. By excluding individuals with completely normal function (MBI = 100), we were able to observe more clearly the intrinsic patterns of functional impairment, particularly identifying the critical age points at which different functional domains began to show significant decline. After the deduplication process, each participant contributed exactly one observation to the final analytical sample.

### Participant selection process

**Fig. 1:**
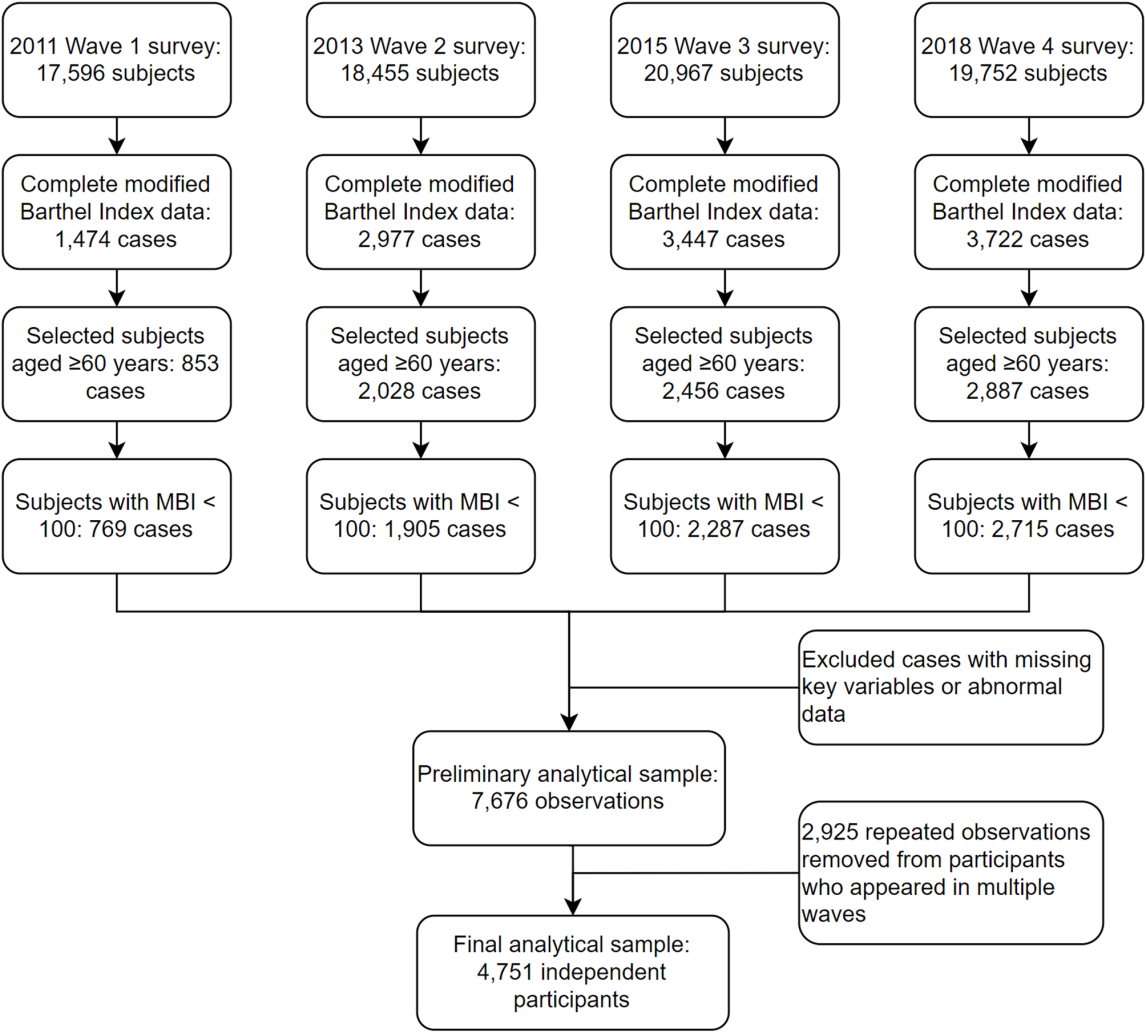
Participant Selection Flowchart.

This flowchart(Fig. 1) illustrates the participant selection process from four CHARLS survey waves (2011, 2013, 2015, 2018). Starting from 76,770 total observations, participants were selected based on complete Modified Barthel Index data, age ≥ 60 years, and functional impairment (MBI < 100), yielding 7,676 observations from 4,751 unique individuals. To ensure statistical independence, a random deduplication process was implemented for participants appearing in multiple waves, resulting in a final analytical sample of 4,751 independent participants.

### Age stratification design

To systematically assess age-related patterns of differences in activities of daily living, we employed an equal-interval age stratification strategy, categorizing participants into seven 5-year age groups: 60–64, 65–69, 70–74, 75–79, 80–84, 85–89, and ≥ 90 years. The final sample sizes for each age group were: 1,213 (60–64 years), 1,052 (65–69 years), 895 (70–74 years), 735 (75–79 years), 507 (80–84 years), 249 (85–89 years), and 100 (≥ 90 years).

The 5-year grouping interval was selected based on a systematic comparative analysis of different grouping ranges (2–10 years), which evaluated statistical significance, detection sensitivity, sample size adequacy, and clinical utility. The 5-year scheme achieved optimal balance between statistical power and preservation of critical stage characteristics of functional changes (see appendix table A1: “Age Stratification Design” for detailed comparison).

### Outcome measures

The primary outcome measures were derived from the Modified Barthel Index (MBI). The MBI is a validated, widely utilized instrument for assessing activities of daily living (ADL) and functional independence^10^. For this study, we analyzed 12 ADL components assessed in CHARLS: eating, bathing, grooming, dressing, toileting, urination control, defecation control, bed/chair transfer, stair climbing, getting up, walking, and wheelchair use. Each activity was rated on a four-level scale reflecting increasing difficulty. These categorical responses were converted into quantitative scores, with higher values indicating greater independence (see Table 1 for scoring details).

Notably, wheelchair use showed extremely low prevalence across all age groups in our community-dwelling sample, with fewer than 5% of participants reporting wheelchair utilization in any age group. Given this limited sample size and the resulting low statistical power, wheelchair use was excluded from detailed statistical analyses and interpretation. Therefore, our primary analysis focused on the remaining 11 MBI domains that demonstrated adequate sample sizes for robust statistical evaluation.

**Table 1:** Scoring criteria for each modified Barthel index (MBI) score.

MBI Item (Activity)	Difficulty Level (Response Options)	Score
【Eating】Because of health and memory problems, do you have any difficulty with eating, such as cutting up your food? (Definition: By eating, we mean eating food by oneself when it is ready.)	(1)No, I don’t have any difficulty	10
	(2)I have difficulty but can still do it.	5
	(3)Yes, I have difficulty and need help.	2
	(4)I can not do it.	0
【Bathing】Because of health and memory problems, do you have any difficulty with bathing or showering?	(1)No, I don’t have any difficulty	5
	(2)I have difficulty but can still do it.	3
	(3)Yes, I have difficulty and need help.	1
	(4)I can not do it.	0
【Grooming】Because of health and memory problems, do you have any difficulties with doing household chores? (Definition: By doing household chores, we mean house cleaning, doing dishes, making the bed, and arranging the house.)	(1)No, I don’t have any difficulty	5
	(2)I have difficulty but can still do it.	3
	(3)Yes, I have difficulty and need help.	1
	(4)I can not do it	0
【Dressing】Because of health and memory problems, do you have any difficulty with dressing? Dressing includes taking clothes out from a closet, putting them on, buttoning up, and fastening a belt.	(1)No, I don’t have any difficulty	10
	(2)I have difficulty but can still do it.	5
	(3)Yes, I have difficulty and need help.	2
	(4)I can not do it	0
【Toileting】Because of health and memory problems, do you have any difficulties with using the toilet, including getting up and down?	(1)No, I don’t have any difficulty	10
	(2)I have difficulty but can still do it.	5
	(3)Yes, I have difficulty and need help.	2
	(4)I can not do it.	0
【Urination Control/Defecation Control】Because of health and memory problems, do you have any difficulties with controlling urination and defecation? If you use a catheter (conduit) or a pouch by yourself, then you are not considered to have difficulties.	(1)No, I don’t have any difficulty	10
	(2)I have difficulty but can still do it.	5
	(3)Yes, I have difficulty and need help.	2
	(4)I can not do it	0
【Bed/Chair Transfer】Do you have any difficulty with getting into or out of bed?	(1)No, I don’t have any difficulty	15
	(2)I have difficulty but can still do it.	8
	(3)Yes, I have difficulty and need help.	3
	(4)I can not do it	0
【Stair Climbing】Do you have difficulty …Climbing several flights of stairs without resting…?	(1)No, I don’t have any difficulty	10
	(2)I have difficulty but can still do it.	5
	(3)Yes, I have difficulty and need help.	2
	(4)I can not do it	0
【Getting Up】Do you have difficulty …Getting up from a chair after sitting for a long period…?	(1)No, I don’t have any difficulty	5
	(2)I have difficulty but can still do it.	3
	(3)Yes, I have difficulty and need help.	1
	(4)I can not do it.	0
【Walking】Do you have difficulty … Walking 100 m…?	(1)No, I don’t have any difficulty	15
	(2)I have difficulty but can still do it.	12
	(3)Yes, I have difficulty and need help.	8
	(4) Unable to complete and the previous question’s selection is 1–3	2
	(5) Unable to complete and the previous question’s selection is 4	0
【Wheelchair Use】Do you use the following auxiliary? (Options 3 or 4 are considered able to operate a wheelchair.)	(3) Manual wheelchair	5
	(4) Electric Wheelchair	5
	Other options	0

Each MBI domain includes multiple response options reflecting the individual’s level of difficulty in performing the activity due to health or memory problems. Higher scores indicate greater independence, whereas lower scores indicate a higher degree of assistance needed. For each activity, the first response option (No difficulty) yields the highest score, and subsequent response options reflect increasing levels of difficulty and lower scores.

### Scale reliability and validity

The Chinese version of the Modified Barthel Index (MBI) used in this study has been validated for reliability and validity. According to relevant studies, the simplified Chinese version of MBI demonstrates excellent psychometric properties. In terms of reliability, the scale shows high internal consistency (Cronbach’s α = 0.929)^11^, inter-rater reliability (ICC = 0.880 ~ 0.992), and test-retest reliability (ICC = 0.909 ~ 0.991)^12^. Regarding validity, the scale has good content validity (mean CVI = 0.95) and criterion validity, with a Spearman correlation coefficient of 0.941 when compared to the original Barthel Index, indicating high consistency between the two.

### Covariates

We included demographic variables (age, sex, marital status), lifestyle factors (smoking status, alcohol consumption), physical and anthropometric measures (height, weight, waist circumference, body mass index [BMI], and systolic and diastolic blood pressure [SBP, DBP]), and physical functioning measures (grip strength, time required to complete five chair stands, and number of completed chair stands). Psychological status was assessed using via a single-item loneliness question derived from the Center for Epidemiologic Studies Depression Scale (CES-D)^13,14^.

### Sample representativeness

The CHARLS dataset used in this study employed a scientifically rigorous multistage stratified probability sampling design to ensure the sample represented the true situation of China’s middle-aged and elderly population. The survey used a multistage probability-proportional-to-size sampling technique, randomly selecting 150 county-level units covering 28 provinces across the country. The sample was stratified by region, urban-rural division, and level of economic development to ensure geographical and socioeconomic representativeness^9^. Our random sampling approach for handling repeated measurements preserved this representativeness while ensuring statistical independence. Given that CHARLS’s sampling design already ensured national representativeness, the age group distribution in our final sample of 4,751 participants can be considered to reflect the true age structure of China’s functionally impaired elderly population.

### Statistical analysis

All statistical analyses were conducted using R software, version 4.4.1 (R Foundation for Statistical Computing, Vienna, Austria). Descriptive statistics are expressed as means and standard deviations (SDs) for continuous variables and as frequencies and percentages for categorical variables.

Given that our deduplication process ensured that each participant contributed exactly one observation, the dataset satisfied the independence assumption required for conventional statistical methods. We employed simple linear models and independent sample t-tests for group comparisons, calculating point estimates and 95% confidence intervals for differences between age groups.

For each MBI domain, we fit linear models with age group as the primary predictor: Score ~ Age_Group. Estimated marginal means were calculated using the emmeans package, and contrast analyses were conducted to examine differences between adjacent age groups, with significance level set at *p* < 0.05. We examined differences in MBI domain scores across adjacent 5-year age groups using pairwise comparisons. This comparative approach highlighted incremental changes between consecutive age categories, allowing for the identification of critical transitional periods in functional decline.

Gender-stratified analyses were performed by calculating descriptive statistics separately for males and females within each age group, followed by gender comparisons within each age group using independent sample t-tests. All statistical results report point estimates, 95% confidence intervals, and p-values, with emphasis on effect sizes and clinical significance rather than statistical significance alone.

Data visualization was conducted using the ggplot2 package, generating bar charts displaying mean MBI domain scores with error bars (± 1 standard deviation) across age groups. Each age group was represented by a distinct color to facilitate visual comparison. Statistical significance markers (* *p* < 0.05, ** *p* < 0.01, *** *p* < 0.001) were added above the bars to highlight significant differences between adjacent age groups.

### Ethical considerations

All participants provided written informed consent at the time of enrollment in CHARLS. The CHARLS study protocol received approval from the Biomedical Ethics Committee of Peking University and adhered to the principles outlined in the Declaration of Helsinki. Our study followed the Strengthening the Reporting of Observational Studies in Epidemiology (STROBE) guidelines for reporting cross-sectional data.

**Table 2:** Baseline characteristics of functionally impaired older adults (*MBI < 100*) across age groups.

Characteristic	60–64	65–69	70–74	75–79	80–84	85–89	90 and above	*P* value
Participants, No	1213	1052	895	735	507	249	100	
Gender								
Male	445 (36.7)	387 (36.8)	365 (40.8)	332 (45.2)	214 (42.2)	104 (41.8)	29 (29.0)	< 0.001
Female	768 (63.3)	665 (63.2)	530 (59.2)	403 (54.8)	293 (57.8)	145 (58.2)	71 (71.0)	
Marital Status								
Yes	982 (81.0)	812 (77.2)	614 (68.6)	421 (57.3)	241 (47.5)	92 (36.9)	14 (14.0)	< 0.001
No	231 (19.0)	240 (22.8)	281 (31.4)	314 (42.7)	266 (52.5)	157 (63.1)	86 (86.0)	
Smoking Status								
Yes	453 (37.3)	386 (36.7)	374 (41.8)	341 (46.4)	217 (42.8)	104 (41.8)	32 (32.0)	< 0.001
No	760 (62.7)	666 (63.3)	520 (58.2)	394 (53.6)	290 (57.2)	145 (58.2)	68 (68.0)	
Alcohol Consumption								
Yes	457 (37.7)	393 (37.4)	389 (43.6)	291 (39.7)	182 (36.0)	87 (34.9)	24 (24.0)	0.001
No	755 (62.3)	658 (62.6)	504 (56.4)	442 (60.3)	324 (64.0)	162 (65.1)	76 (76.0)	
SBP, mmHg	131.41 (22.21)	134.47 (23.64)	135.14 (23.38)	139.62 (22.77)	141.25 (24.33)	140.61 (25.47)	142.08 (29.25)	< 0.001
DBP, mmHg	77.07 (13.30)	76.83 (13.95)	75.02 (13.27)	74.08 (12.94)	73.01 (12.11)	72.34 (13.78)	74.02 (13.32)	< 0.001
Grip Strength, kg	26.20 (9.93)	24.53 (9.48)	23.57 (8.88)	22.52 (8.37)	19.63 (7.86)	17.71 (8.92)	17.32 (8.08)	< 0.001
Height, m	1.56 (0.08)	1.55 (0.09)	1.53 (0.11)	1.54 (0.10)	1.52 (0.10)	1.50 (0.09)	1.48 (0.08)	< 0.001
Weight, kg	59.18 (11.10)	58.12 (11.86)	54.57 (11.21)	53.14 (12.03)	51.28 (10.67)	46.31 (10.57)	44.80 (8.33)	< 0.001
Waist, cm	86.74 (14.52)	88.36 (12.82)	85.94 (14.44)	86.43 (13.37)	85.94 (14.63)	83.64 (12.72)	81.62 (9.16)	0.019
BMI, kg/m2	24.42 (5.94)	24.03 (4.18)	23.83 (13.53)	22.45 (4.24)	22.22 (3.89)	20.52 (3.30)	20.34 (2.73)	< 0.001
Time for 5 Chair Stands(second)	11.52 (3.90)	12.02 (4.06)	13.41 (5.25)	14.15 (6.29)	15.71 (6.92)	16.90 (7.92)	19.53 (12.98)	< 0.001
Number of Chair Stands Completed	5.00 (0.09)	4.98 (0.21)	4.99 (0.12)	4.99 (0.14)	5.00 (0.00)	5.00 (0.00)	5.00 (0.00)	0.902
CES-D score	12.72 (7.22)	11.75 (6.97)	11.71 (6.74)	10.55 (6.67)	10.35 (6.70)	8.88 (5.86)	9.54 (5.30)	< 0.001
Modified Barthel Index	79.37 (18.27)	78.54 (18.88)	76.14 (20.68)	75.08 (21.44)	70.31 (24.15)	69.58 (23.50)	64.91 (25.69)	< 0.001

This table presents the baseline characteristics of the study participants stratified by age groups (60–64, 65–69, 70–74, 75–79, 80–84, 85–89, 90 years and above). Data are expressed as n (%) for categorical variables and means (standard deviations) for continuous variables. *P* values were calculated via chi-square tests for categorical variables and ANOVA for continuous variables. Significant p values (*p* < 0.05) indicate significant differences across age groups. BMI, calculated as weight in kilograms divided by height in meters squared.

## Results

### Baseline characteristics

After random deduplication, a total of 4,751 independent participants aged 60 years and older were included in the final analysis. Table 2 summarizes the baseline characteristics stratified by seven five-year age groups (60–64, 65–69, 70–74, 75–79, 80–84, 85–89, and ≥ 90 years). The sample sizes for each age group were: 1,213 (60–64 years), 1,052 (65–69 years), 895 (70–74 years), 735 (75–79 years), 507 (80–84 years), 249 (85–89 years), and 100 (≥ 90 years).

Demographic and lifestyle factors demonstrated significant age-related variations. The proportion of male participants increased from 36.7% in the 60–64 years group to 45.2% in the 75–79 years group, then decreased to 29.0% in the ≥ 90 years group (*p* < 0.001). Marital status showed a pronounced decline with age: the percentage of married individuals decreased dramatically from 81.0% at ages 60–64 to only 14.0% at ages ≥ 90 (*p* < 0.001). Smoking prevalence varied significantly across age groups, with the highest rates observed in the 75–79 years group (46.4%) and the lowest in the ≥ 90 years group (32.0%, *p* < 0.001). Alcohol consumption peaked in the 70–74 years group (43.6%) and declined substantially in older age groups, reaching 24.0% in the ≥ 90 years group (*p* = 0.001).

Physical and anthropometric measures revealed clear age-related trajectories. Systolic blood pressure (SBP) showed a consistent upward trend from 131.41 mmHg in the 60–64 years group to 142.08 mmHg in the ≥ 90 years group (*p* < 0.001), while diastolic blood pressure (DBP) generally decreased from 77.07 mmHg to 74.02 mmHg across the same age range (*p* < 0.001). Body composition changes were evident, with height decreasing from 1.56 m to 1.48 m (*p* < 0.001), weight declining from 59.18 kg to 44.80 kg (*p* < 0.001), and BMI dropping from 24.42 kg/m² to 20.34 kg/m² (*p* < 0.001) across the age spectrum. Waist circumference showed a modest but significant decrease from 86.74 cm to 81.62 cm (*p* = 0.019).

Functional capacity measures demonstrated substantial age-related decline. Grip strength decreased markedly from 26.20 kg in the 60–64 years group to 17.32 kg in the ≥ 90 years group (*p* < 0.001), representing a 34% decline. The time required to complete five chair stands increased progressively from 11.52 s to 19.53 s across age groups (*p* < 0.001), indicating significant deterioration in lower-extremity function. However, the number of completed chair stands remained relatively stable across all age groups (*p* = 0.902).

Psychological well-being, assessed by depression scale scores, showed an interesting pattern: despite expectations of increased depression with age, scores actually decreased from 12.72 in the youngest group to 9.54 in the oldest group (*p* < 0.001), suggesting potentially better psychological adjustment or survivorship effects in the oldest participants. The overall Modified Barthel Index scores confirmed the expected pattern of functional decline, decreasing from 79.37 in the 60–64 years group to 64.91 in the ≥ 90 years group (*p* < 0.001).

### Overall ADL differences and domain-specific changes

**Fig. 2:**
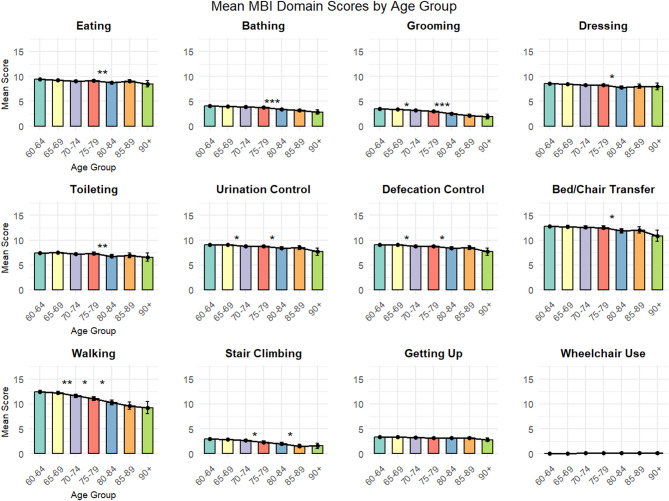
Mean MBI Domain Scores by Age Group. The bars represent mean Modified Barthel Index (MBI) scores for each of the 12 functional domains across seven age groups, with error bars indicating ± 1 standard deviation. Statistical significance between adjacent age groups was determined using simple linear models, with pairwise comparisons conducted using estimated marginal means. Significance levels are indicated as * *p* < 0.05, ** *p* < 0.01, and *** *p* < 0.001. Higher scores indicate greater functional independence.

Among the 12 MBI domains assessed, 11 domains were included in the detailed statistical analysis. Wheelchair use was excluded due to extremely low prevalence (< 5% across all age groups), which precluded meaningful statistical interpretation(Detailed descriptive statistics are presented in Supplementary Table S1). Using simple linear models to analyze age-related differences across 11 MBI domains, we conducted 72 pairwise comparisons between adjacent age groups, of which 17 comparisons (23.6%) achieved statistical significance(Fig. 2). (Comprehensive results of all age group comparisons are detailed in Supplementary Table S2)The analysis revealed three distinct patterns of functional change across age groups:

### Pattern I: relatively stable basic self-care functions

Eating ability demonstrated remarkable stability across most age transitions, with only one statistically significant change occurring between the 75–79 and 80–84 years age groups (difference = 0.40, *p* = 0.0095), representing a modest improvement rather than decline. Getting up ability showed the greatest stability among all functions, with no statistically significant differences detected across any adjacent age group comparisons, suggesting this basic transfer skill is well-preserved even in the presence of functional impairment.

### Pattern II: the critical 75–84 years transition period

The 75–79 to 80–84 years age transition emerged as a critical inflection point for multiple functional domains. Bathing ability showed highly significant decline during this period (difference = 0.427, *p* < 0.001), representing one of the most pronounced single-transition deteriorations observed. Toileting ability similarly demonstrated significant decline (difference = 0.597, *p* = 0.0043), as did bed/chair transfer ability (difference = 0.634, *p* = 0.0198). Dressing ability also showed significant deterioration during this transition (difference = 0.475, *p* = 0.0136).

Excretory control functions (both urination and defecation control) exhibited a distinctive two-phase decline pattern. Initial significant deterioration occurred between 65 and 69 and 70–74 years (difference = 0.302, *p* = 0.0116), followed by a second significant decline during the critical 75–79 to 80–84 years transition (difference = 0.347, *p* = 0.0491).

### Pattern III: continuous decline in high-demand activities

Walking ability displayed the most extensive pattern of continuous functional decline, showing significant deterioration across three consecutive age transitions: 65–69 to 70–74 years (difference = 0.622, *p* = 0.0058), 70–74 to 75–79 years (difference = 0.533, *p* = 0.0442), and 75–79 to 80–84 years (difference = 0.793, *p* = 0.0136). This continuous decline pattern was unique among all MBI domains, suggesting walking ability may serve as an early indicator of overall functional deterioration.

Grooming ability showed significant decline beginning in the 65–69 to 70–74 years transition (difference = 0.196, *p* = 0.0335), with highly significant deterioration during the critical 75–79 to 80–84 years period (difference = 0.492, *p* < 0.001).

Stair climbing ability demonstrated an intermittent decline pattern, with significant deterioration occurring during two distinct periods: 70–74 to 75–79 years (difference = 0.327, *p* = 0.0249) and 80–84 to 85–89 years (difference = 0.488, *p* = 0.0188).

### Gender-stratified analysis

**Fig. 3:**
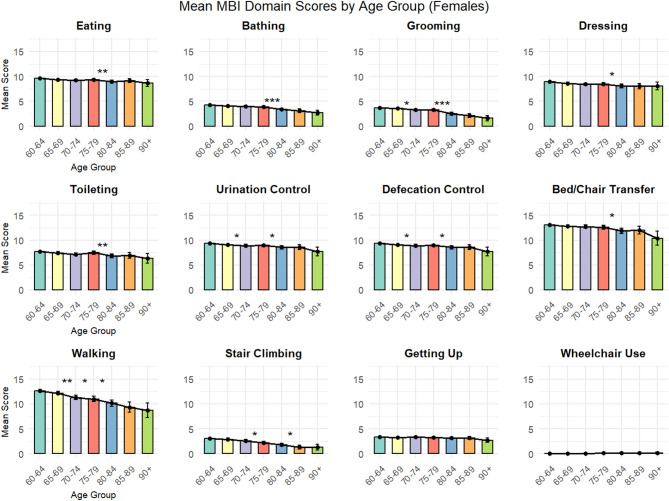
Mean MBI Domain Scores by Age Group for Females.

**Fig. 4:**
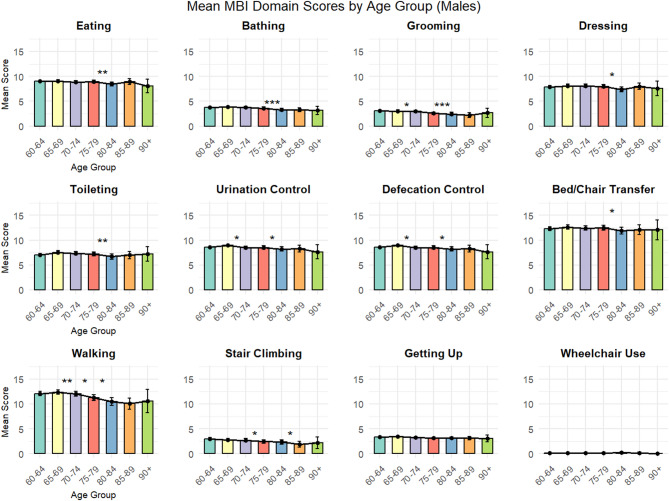
Mean MBI Domain Scores by Age Group (Males). The bars represent mean Modified Barthel Index (MBI) scores for each functional domain across seven age groups, stratified by gender, with error bars indicating ± 1 standard deviation. Statistical significance between adjacent age groups is indicated as * *p* < 0.05, ** *p* < 0.01, and *** *p* < 0.001. Higher scores indicate greater functional independence.

Gender-stratified analysis revealed significant sex-specific patterns across multiple functional domains(Figs. 3 and 4). (Detailed gender-stratified statistics by age group are provided in Supplementary Table S3). In basic self-care abilities, females generally demonstrated superior functional performance across most age groups.

Eating ability showed the largest gender difference in the 60–64 years age group, with males scoring 0.55 points lower than females (*p* < 0.001).(Complete results of gender comparisons across all functional domains are presented in Supplementary Table S4) This gender gap diminished with age, becoming non-significant in most older age groups.

Grooming ability demonstrated persistent gender differences favoring females, with significant differences observed in the 60–64 years (difference = 0.62, *p* < 0.001), 65–69 years (difference = 0.539, *p* < 0.001), and 75–79 years (difference = 0.699, *p* < 0.001) age groups.

Dressing ability showed the most pronounced gender differences in younger elderly groups, with the largest difference in the 60–64 years group (difference = 1.075, *p* < 0.001). This gender advantage for females gradually diminished with advancing age.

Walking ability revealed an intriguing age-related gender reversal pattern. In the 60–64 years group, females demonstrated superior walking ability (males scored 0.584 points lower, *p* = 0.0345). However, by the 70–74 years group, this pattern reversed, with males showing better walking performance (males scored 0.766 points higher, *p* = 0.0268).

Excretory control functions (urination and defecation control) showed consistent gender differences favoring females in younger age groups, particularly pronounced in the 60–64 years group (difference = 0.835, *p* < 0.001), with differences diminishing in older age groups.

Notably, several functional domains showed minimal gender differences across age groups, including toileting, bed/chair transfer, getting up, and stair climbing, suggesting that age-related changes in these functions follow similar patterns in both sexes.

### Summary of key findings

This comprehensive analysis identified the 75–79 to 80–84 years age transition as a critical period for functional decline, with 6 out of 12 MBI domains showing significant deterioration during this interval. Walking ability emerged as the most vulnerable function, demonstrating continuous decline across multiple age transitions and potentially serving as an early warning indicator for overall functional deterioration. Basic self-care functions such as eating and getting up showed remarkable resilience across age groups. Gender analysis revealed female advantages in basic self-care abilities that gradually diminish with age, and an intriguing reversal in walking ability gender differences occurring in the early 70 s age range.

## Discussion

This study reveals distinct age-related patterns of functional differences in activities of daily living among community-dwelling older Chinese adults. Our findings demonstrate a tiered pattern of functional decline, where basic self-care activities remain relatively stable while more complex tasks show earlier and more pronounced deterioration^15^. Most significantly, the 75–79 to 80–84 years transition emerged as the critical inflection point for functional decline, with 6 out of 12 MBI domains showing significant deterioration during this specific interval.

Our findings underscore that certain core self-care activities, particularly getting up ability, demonstrated remarkable stability with no significant changes across any age transitions. Eating ability similarly showed minimal variation, reflecting this task’s reliance on ingrained, habitual behaviors and lower physical and cognitive demands^16^. This resilience suggests that basic self-maintenance capabilities are well-preserved even among functionally impaired older adults, though ongoing surveillance remains important as individuals may still be vulnerable to sudden health events^17^.

Walking ability emerged as the most vulnerable function, showing unique continuous decline across three consecutive age transitions (65–69 to 70–74, 70–74 to 75–79, and 75–79 to 80–84 years). This pattern distinguishes walking from all other functional domains and supports its potential role as an early warning indicator for overall functional deterioration^18^. The comprehensive nature of walking—requiring integration of musculoskeletal, neurological, and sensory systems—likely explains its early and sustained vulnerability^19^. The concentration of functional decline during the 75–84 years transition period represents a significant departure from previous research emphasizing earlier critical periods^20–22^. This finding suggests that targeted interventions may demonstrate optimal efficacy when targeted at individuals approaching their late 70 s, rather than focusing exclusively on the traditional 65–75 years age window. The delayed onset of major functional deterioration may reflect improvements in healthcare and nutrition among recent elderly cohorts in China^23^.

Gender-stratified analysis revealed intriguing sex-specific patterns, particularly the age-related reversal in walking ability differences. While females demonstrated superior walking performance in the 60–64 years group, this advantage reversed by the 70–74 years group, with males showing better performance^24^. This pattern may be attributed to post-menopausal hormonal changes affecting muscle mass and bone density in females^25,26^, combined with sociocultural influences related to traditional gender roles in China^27^.

Our methodological approach of random sampling to ensure statistical independence represents an important advancement over traditional mixed-effects modeling approaches^28^. While this strategy reduced sample size by 38%, it eliminated statistical dependency and enhanced the validity of our findings. The emphasis on effect sizes and confidence intervals rather than mere statistical significance provides more clinically meaningful information for intervention planning^29^.

These results extend the literature by identifying age-specific critical periods that differ from international patterns, highlighting the importance of population-specific research for intervention planning. The findings suggest that clinicians and policy-makers should prioritize walking ability preservation as a key strategy for preventing broader functional decline, while preparing intensive support programs for individuals entering their late 70s^30^.

These results align with extant evidence that complex motor tasks are among the first to exhibit deterioration in older adults^31^, underscoring the interplay of musculoskeletal, neurological, and sensory factors in functional aging^32^. The detection of mild but significant declines in functions such as getting up and controlling defecation at a slightly earlier juncture highlights how even subtle changes may presage more substantial impairments over time^33^. Recognizing these early warning signs is essential for timely, targeted interventions^34^.

## Limitations

Several limitations should be acknowledged. The random deduplication process reduced sample size by 38% (from 7,676 to 4,751 participants), potentially affecting statistical power, particularly in the oldest age group (≥ 90 years, *n* = 100). However, ensuring statistical independence was essential for valid inferences.

The cross-sectional design precludes causal inferences, as observed differences may reflect cohort effects or survivorship bias rather than pure age effects. Survivorship bias may be particularly relevant in older age groups, as participants likely represent relatively healthy survivors rather than typical representatives of their birth cohorts.

Reliance on self-reported data may introduce reporting biases influenced by cognitive ability and psychological state. Future research should incorporate objective, performance-based assessments for more reliable functional evaluation.

Including only older adults with MBI < 100 limits generalizability to the broader elderly population, while unmeasured confounding variables such as socioeconomic status, chronic disease burden, and social support networks may influence patterns of ADL decline. Longitudinal studies are needed to confirm these age-related patterns and capture individual-level changes over time.

## Conclusion

This study identified distinct patterns of age-related differences in activities of daily living among 4,751 independent older Chinese adults, with basic self-care abilities (eating, getting up) remaining remarkably stable while walking ability showed continuous decline across multiple age transitions. The 75–79 to 80–84 years transition emerges as a critical period for functional decline, with six functional domains showing significant deterioration during this interval, suggesting that preventive interventions should target this later age window to effectively delay functional decline and preserve independence in older adults.

## Supplementary Information

Below is the link to the electronic supplementary material.

Supplementary Material 1

Supplementary Material 2

Supplementary Material 3

Supplementary Material 4

Supplementary Material 5

## Data Availability

The data used in the present study were obtained from publicly accessible sources. The China Health and Retirement Longitudinal Study (CHARLS) dataset, described in this study, is publicly available at the CHARLS website: https://charls.pku.edu.cn/.
