# Supplementary material for: Age-related differences in activities of daily living among older Chinese adults
Source: Sci Rep. 2025 Oct 3;15:34604. doi: 10.1038/s41598-025-18163-y (PMC12494767; doi:10.1038/s41598-025-18163-y)
Supplement: Supplementary file 1 — Supplementary Material 1 [file 41598_2025_18163_MOESM1_ESM.docx]

Table A1. Comparison of Statistical Metrics Across Different Age Grouping Intervals

| Age Group (years) | Avg ANOVA p-value | Total Significant Differences |
| --- | --- | --- |
| 2 | 0.008836653 | 343 |
| 3 | 0.00296299 | 243 |
| 4 | 0.0012674 | 189 |
| 5 | 0.000650022 | 138 |
| 6 | 0.001390767 | 114 |
| 7 | 0.004326687 | 93 |
| 8 | 0.000549852 | 84 |
| 9 | 0.001345657 | 67 |
| 10 | 0.000224993 | 65 |

Comparison results showed that the 5-year grouping scheme achieved the optimal balance between average ANOVA P-value (0.0006) and total number of significant differences (138). Compared to narrower grouping intervals (2, 3 years), the 5-year grouping avoided excessive subdivision leading to insufficient sample size and reduced statistical power; compared to wider grouping intervals (8, 10 years), the 5-year grouping preserved critical stage characteristics of functional changes. Additionally, the 5-year grouping offered intuitive and practical clinical application, facilitating translation into intervention strategies and policy recommendations.
